# Nitric Oxide-Mediated Posttranslational Modifications: Impacts at the Synapse

**DOI:** 10.1155/2016/5681036

**Published:** 2015-11-09

**Authors:** Sophie A. Bradley, Joern R. Steinert

**Affiliations:** MRC Toxicology Unit, Hodgkin Building, Leicester LE1 9HN, UK

## Abstract

Nitric oxide (NO) is an important gasotransmitter molecule that is involved in numerous physiological processes throughout the nervous system. In addition to its involvement in physiological plasticity processes (long-term potentiation, LTP; long-term depression, LTD) which can include NMDAR-mediated calcium-dependent activation of neuronal nitric oxide synthase (nNOS), new insights into physiological and pathological consequences of nitrergic signalling have recently emerged. In addition to the canonical cGMP-mediated signalling, NO is also implicated in numerous pathways involving posttranslational modifications. In this review we discuss the multiple effects of S-nitrosylation and 3-nitrotyrosination on proteins with potential modulation of function but limit the analyses to signalling involved in synaptic transmission and vesicular release. Here, crucial proteins which mediate synaptic transmission can undergo posttranslational modifications with either pre- or postsynaptic origin. During normal brain function, both pathways serve as important cellular signalling cascades that modulate a diverse array of physiological processes, including synaptic plasticity, transcriptional activity, and neuronal survival. In contrast, evidence suggests that aging and disease can induce nitrosative stress *via* excessive NO production. Consequently, uncontrolled S-nitrosylation/3-nitrotyrosination can occur and represent pathological features that contribute to the onset and progression of various neurodegenerative diseases, including Parkinson's, Alzheimer's, and Huntington's.

## 1. Introduction

Since its characterisation in the early 1980s by Furchgott, Ignarro and others [1–3], nitric oxide (NO) has been widely recognised as an important signalling molecule in many physiological processes. The initial identification of NO as endothelium-derived relaxing factor (EDRF) [4] generated a great interest in its function in vascular biology. Over subsequent years, the focus on NO research rapidly expanded from the vascular system to its role in immunity and inflammation, cell death, cell survival, and aging, to name but a few. Of particular interest is its role within the nervous system and its function in neuronal signalling. NO was first identified to be present in the central nervous system by the discovery of one of its synthesising enzymes, neuronal nitric oxide synthase (nNOS), within the mammalian brain [5]. Aside from its production through nNOS, NO can also be synthesised through activation of either one of the two other nitric oxide synthases termed endothelial nitric oxide synthase (eNOS) and inducible nitric oxide synthase (iNOS) [6]. After synthesis, NO can bind to its predominant physiological receptor soluble guanylyl cyclase (sGC) to catalyse the conversion of guanosine-5′-triphosphate (GTP) to cyclic guanosine monophosphate (cGMP). From here cGMP can regulate the activity of many downstream targets such as the modulation of protein kinases and ion channels, demonstrating that relatively low amounts of generated NO can be amplified substantially through this signalling pathway. Following the initial characterisation of NO, its diverse function was soon recognized throughout the nervous system [7]. NO generation* via* nNOS in response to NMDAR activation was one of the earliest pathways characterised in the brain [7, 8] and it became evident that NO could serve as an important signalling molecule within neurons. Involvement of NO ranges from synaptic plasticity and activity modulation [9], such as LTP/LTD, to pathological actions seen in many neurodegenerative conditions [10].

**Figure 1:**
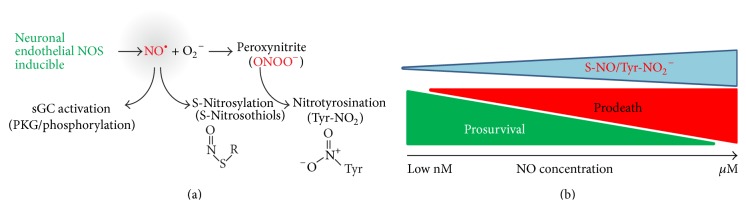
Nitric oxide profile and posttranslational modifications. This figure indicates pathways of NO generation and posttranslational modifications. (a) Generation of NO by the three different NO synthases leads to activation of the sGC and thiol nitrosylation forming S-nitrosothiols. Further reaction of NO with oxygen radicals leads to the formation of peroxynitrite and subsequent irreversible modification of tyrosine residues. (b) Concentration dependency between NO levels and the amount of posttranslational modifications with associated dominance of prosurvival or prodeath signalling.

It is now generally acknowledged that, in addition to the canonical sGC/cGMP pathway mentioned above, NO has additional roles in modulating protein function* via* induction of posttranslational modifications. NO can lead to thiol nitrosylation of cysteine residues termed S-nitrosylation (–SNO, covalent and reversible attachment of an NO molecule to a thiol group [–SH]) and tyrosine nitration termed 3-nitrotyrosination (NO_2_-Tyr* via* peroxynitrite formation [ONOO^−^], Figure 1). These modifications impact on protein-protein interactions, protein structure, and function and are largely generated through the excessive production of NO which occurs through overactivation of nNOS or induction of iNOS* via* neuroinflammatory stimuli or additional toxins. Although S-nitrosylation is an important modulator of protein function under physiological conditions, it is largely detrimental under pathophysiological conditions due to the high levels of reactive oxygen species and reactive nitrogen species present. Similarly, tyrosine nitration is predominantly damaging due to its occurrence in environments where toxic peroxynitrite is generated. An important and differing characteristic of the two processes is that S-nitrosylation is a reversible mechanism, the equilibrium of which can be shifted by the activities of reductases, namely, thioredoxin or S-nitrosoglutathione reductase [11, 12], whereas 3-nitrotyrosination is an irreversible modification. Furthermore, the equilibrium between nitrosylation and denitrosylation can be differentially affected during disease and aging which may then further perpetuate these processes making it an important signalling pathway in physiology and pathology.

The above modifications have been implicated in many cellular processes, such as modulation of transcription factors, membrane receptors, and general effects on neuronal development, health, and survival or differentiation [10, 11, 13–16]. The mechanisms by which nitrergic activity can regulate gene expression and thereby determine the fate of a neuron can be widespread [17]; however, this review focuses specifically on direct nitrergic effects related to synaptic function and transmitter release.

As the likelihood of S-nitrosylation increases in a hydrophobic environment [11, 12], proteins attached to the membrane or localized within cellular membrane microdomains are highly susceptible to these modifications. This therefore applies not only, for instance, to postsynaptic density proteins and neurotransmitter receptors, but also to membrane-associated molecules of the presynaptic release machinery. Although NO targets include ion channels, which are involved in setting neuronal excitability and calcium homeostasis [18–23] and are thereby indirectly involved in synaptic function, we do not cover this area as this topic has been reviewed elsewhere [24, 25].

## 2. Impacts of NO Signalling at the Synapse

Glutamate is the major excitatory neurotransmitter in the mammalian central nervous system and its downstream functions are mediated by metabotropic and ionotropic glutamate receptors. Several groups have reported nitrergic modulation of glutamate signalling. It has been shown that NO was able to stimulate glutamate release in hippocampal slices [26] and increase glutamate release in the rat and mouse hippocampus or rat dorsomedial medulla oblongata [27–29]. However, the molecular mechanisms responsible for these nitrergic modifications are not fully understood and in addition to the canonical pathways posttranslational modifications at the synaptic level may explain some of the above findings [30].

The release of GABA, an inhibitory neurotransmitter, has been shown to be negatively affected by NO in the internal granule cell layer of the cerebellum [31] and in auditory cortical neurons [32]. However, NO can induce an increase in GABA release, as systematically found in other parts of the brain [33]. Furthermore, it has been shown that NO can regulate GABA release differentially in a concentration-dependent manner. There is evidence that basal or low NO concentrations decrease GABA release, whereas high NO concentrations could augment GABA release [34, 35]. In the other brain regions, data are inconsistent with NO mediating an increased GABA release from synaptic vesicles into the cytosol rendering vesicles to be GABA depleted [36], while, in other studies, NO,* via* peroxynitrite formation, induced a potentiation of evoked GABA release [37], and nNOS inhibition resulted in enhanced GABAergic inputs into CA1 pyramidal neurons [38]. These unexplained discrepancies in nitrergic effects are most likely due to its multiple modes of action depending on the cellular environment and illustrate the broad range of NO signalling pathways.

Dopamine (DA) is a neurotransmitter present at high concentrations in specific brain regions of the central nervous system, that is, in the mesencephalon (substantia nigra) [39], and NO-mediated enhanced DA release was initially reported* in vitro* and* in vivo* in the striatum [40, 41]. However, in the intact bovine retina, NO significantly decreased basal or potassium-induced DA release, while NOS inhibition stimulated basal release of DA [42]. As a decrease in DA release is a key contributor to Parkinson's disease, the link between oxidative stress/NO/ONOO^−^ and deficient DA release could relate nitrergic posttranslational modifications to the pathology observed in this disease.

Many of the above data suggest that NO can bidirectionally, possibly in a concentration-dependent manner, regulate transmitter release and postsynaptic responsiveness. Although some of these data indicate cGMP involvement, this is not always the case and posttranslational modifications, as investigated in more recent years, could well explain the observed discrepancies. We discuss below some specific synaptic nitrergic effects related to overall transmitter release and neuronal communication.

### 2.1. Presynapse: NO at the Synaptic Release Machinery

**Figure 2:**
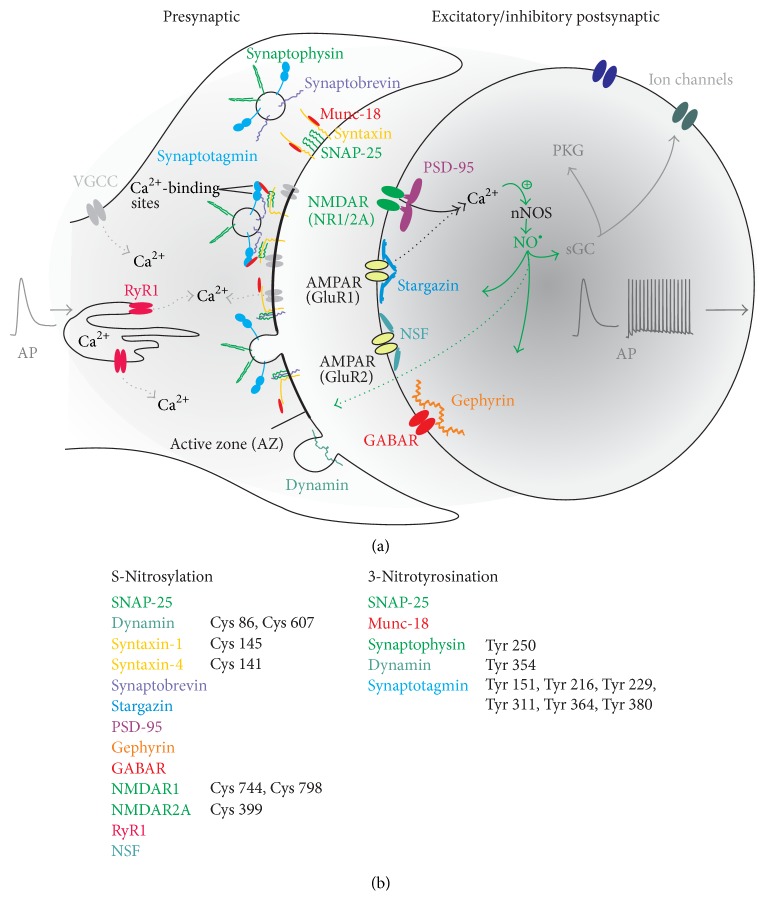
Simplified pathways of synaptic transmission and effects of nitrergic posttranslational modifications. (a) Left, presynapse; right, postsynapse: showing signalling steps involved in transmitter release and neurotransmitter receptor function. Ca^2+^-dependent activation of postsynaptic nNOS leads to nitrergic modifications of molecules (colour-coded) which are involved in synaptic transmission in pre- and postsynaptic compartments. NO diffuses within the postsynaptic cell but also into the presynapse. Presynaptic assembly of the prefusion SNARE complex (such as the vesicular v-SNARE complex protein synaptobrevin assemblies with the plasma membrane SNARE proteins syntaxin and SNAP-25) and membrane proteins in close proximity to the active zone (AZ) results in a prefusion state of the vesicle. In a further step, Munc-18 associates with syntaxin-1 when syntaxin-1 is in a closed conformation; as syntaxin-1 opens during SNARE complex assembly it enables subsequent vesicle fusion and transmitter release following Ca^2+^ influx. Ca^2+^ release from intracellular stores* via* ryanodine receptors (RyR1) as well as influx through voltage-gated Ca^2+^ channels (VGCC, in grey) in response to incoming action potentials (AP) leads to the accumulation of intracellular Ca^2+^levels and promoting of vesicle fusion. After fusion pore opening, the resulting SNARE complexes are disassembled and vesicles are recycled (dynamin-mediated), refilled with neurotransmitter, and reused for release. Postsynaptic receptors, excitatory NMDAR, and inhibitory GABAR are S-nitrosylated. Furthermore, the scaffolding proteins PSD-95, Stargazin, NSF, and Gephyrin are also nitrosylated with various functional outcomes. Ultimately, these modifications in addition to the canonical sGC/cGMP pathway (in grey) will alter the synaptic response and change AP firing characteristics following nitrergic signalling. (b) All colour-coded proteins have been shown to be subject to nitrergic posttranslational modifications with their specific residues indicated.

The presynaptic site represents a crucial part in synaptic transmission. Here, release of vesicles, either filled with excitatory or inhibitory neurotransmitters, by exocytosis and reuptake* via* endocytosis, is essential for neuronal communication [43–45]. There are numerous presynaptic signalling molecules whose regulation determines the amount and availability of released transmitter [43–46]. In general, Ca^2+^ triggers release in a highly cooperative manner [47] within a few hundred microseconds of an incoming action potential [48]. There are several key steps involved in this process which enable molecular mechanisms to deliver fast vesicle fusion at a synapse (see below); however, due to the complexity and specificity of these mechanisms, any modulation of involved molecules contributing to release, that is, by NO, will affect synaptic vesicular fusion. To name just a few involved in these cascades, the Ca^2+^ sensor synaptotagmin, following Ca^2+^ binding, triggers vesicular release by stimulating its interaction with a core fusion machinery composed of SNARE (soluble* N*-ethylmaleimide sensitive factor attachment protein receptor) and synaptic membrane proteins that mediate fusion during exocytosis (Figure 2). Complexin adaptor proteins assist synaptotagmin by activating and clamping this core fusion mechanism so that synaptic vesicles containing synaptotagmin are positioned at the active zone ready for release. Through the specific binding of these specialised proteins (SNARE and Sec1/Munc-18 proteins) a connection is formed between synaptic vesicles and the synaptic membrane resulting in them being brought to close proximity to one another. This enables both rapid fusion and release of synaptic vesicles [49]. There are two types of SNARE proteins that constitute the core complex and these are known as vesicular v-SNAREs and target t-SNAREs. v-SNAREs, such as synaptobrevin/VAMP, are located largely on the synaptic vesicles, whereas t-SNAREs, such as syntaxin or SNAP-25, are located mainly on the plasma membrane. During fusion both t-SNAREs and v-SNAREs form *α*-helical trans*-*SNARE complex that facilitates the tight association of the two membranes. The three SNARE proteins SNAP-25, syntaxin-1, and synaptobrevin are essential components of the synaptic vesicle fusion machinery [44]. Any modulation of any of these proteins could potentially have dramatic impacts on release. Studies have shown that a lack of synaptobrevin-2 activity in mouse hippocampal knockout neurons resulted in a drastic reduction of evoked release with virtually no ready releasable pool available in these neurons [50]. Similarly, modifications of SNAP-25 either by removing the 9 C-terminal residues (SNAP-25Δ9) or by substituting C-terminus amino acids with more positively charged residues resulted in reduced release frequencies, slower release kinetics, and prolonged fusion pore duration [51] confirming the tight regulatory role of these proteins in vesicle fusion. Additionally mutant mice expressing a constitutively open syntaxin-1, which enhances SNARE complex formation, showed an increased speed of evoked release, enhanced Ca^2+^ affinity of release, and accelerated fusion pore expansion [52]. Fast neurotransmission depends not only on coordinated release but also on replenishment of vesicle pools which depends on endocytotic pathways. One molecule involved in endocytosis is the large GTPase dynamin which induces fission of the vesicle from the plasma membrane as shown by using the temperature-sensitive allele of* dynamin* that blocks all synaptic endocytosis at restrictive temperatures [53, 54]. Below we discuss studies reporting nitrergic modulation of several of the above synaptic molecules; however, the literature only partially offers insights into how posttranslational modifications (PTM) can induce functional changes and are largely observational.

#### 2.1.1. S-Nitrosylation

In an earlier study, S-nitrosylation has been investigated in purified synaptic vesicle fractions and it was found that NO donor application led to widespread thiol modifications of proteins [55]; however, this study did not identify any specific residues but concluded that syntaxin-1, SNAP-25, and synaptobrevin of the SNARE complex of synaptic molecules were affected. One of them, syntaxin, is participating in exocytosis and is believed to be stabilized by the classical binding of Munc-18. This critical binding mode of Munc-18 to the closed conformation of syntaxin has been shown to be disrupted following Cys 145 S-nitrosylation [56], thereby reversing the inhibition and facilitating interaction with the membrane fusion machinery. This provides scope on the possibility of S-nitrosylation forming a regulatory mechanism to modulate this inhibitory interaction and subsequently allowing the binding of syntaxin with other SNARE proteins to induce membrane fusion [56]. Interestingly the sequence surrounding the Cys 145 (syntaxin-1) is highly conserved in all neuronal syntaxins implying a general modulatory function of this modification as shown in adrenal chromaffin cells [56] but also in *β*-cells where S-nitrosylation of Cys 141 (syntaxin-4) augments insulin exocytosis [57].

Another important regulatory protein which is predominantly involved in the scission of newly formed vesicles from the membrane is dynamin. This mechanism is crucially involved in presynaptic endocytosis but can also mediate internalisation of postsynaptic receptors from the membrane, thus regulating vesicular trafficking. S-Nitrosylation of dynamin at Cys 86 and Cys 607 has been shown to inhibit its activity in different cellular environments [58, 59], therefore providing evidence that these posttranslational modifications of dynamin could be modulating membrane trafficking.

A more general way to affect synaptic release is* via* upstream modulation of intracellular Ca^2+^ levels. Here, influx of Ca^2+^ through depolarisation-induced activation of voltage-gated Ca^2+^ channels and Ca^2+^ store release,* via* IP_3_ and ryanodine receptors, provides an important signalling mechanism. Enhanced Ca^2+^ store release* via* ryanodine receptor activation is associated with LTP and it has been shown that S-nitrosylation of the ryanodine receptor 1 enhances Ca^2+^ store release [60]. The authors did not further investigate the roles of presynaptic versus postsynaptic ryanodine receptor modulation; however, they concluded from their data that this posttranslational modification contributes to physiological and pathological signalling at the synapse.

#### 2.1.2. 3-Nitrotyrosination

Although nitrotyrosination has not been widely investigated at a synaptic level, some studies indicate that SNAP-25 undergoes 3-nitrotyrosination. SNAP-25 is a t-SNARE that is involved in the specificity of membrane fusion and directly executes fusion by forming a tight trans-SNARE complex with the v-SNAREs that brings the synaptic vesicle and membranes together. A study by Michela Di Stasi et al. showed that peroxynitrite induced tyrosine nitration of SNAP-25 and Munc-18 [61], two of the major proteins involved in protein-protein interactions in the docking/fusion steps of vesicle release. Another regulator of vesicle release is synaptophysin which is one of the most abundant integral proteins of vesicular membranes and is involved in several steps of synaptic function including synapse formation, biogenesis, and exocytosis/endocytosis of synaptic vesicles [62–64]. Although functional data are lacking, it has been shown that synaptophysin is nitrated (Tyr 250) and the formation of the synaptophysin/dynamin complex is impaired following peroxynitrite exposure [65, 66]. Dynamin I on the other hand is also nitrated on Tyr 354 which possibly interferes with tyrosine phosphorylation and results in further diminished complex formation [65] showing that activity of this molecule is affected by both S-nitrosylation and tyrosine nitration in a negative manner.

A further member of the SNARE protein family is the Ca^2+^ sensor synaptotagmin. Synaptotagmins are transmembrane proteins that have two cytoplasmic C2 Ca^2+^-binding domains, C2A and C2B. There are 16 different isoforms of synaptotagmin and depending on the isoform they are localized to either synaptic/secretory vesicles or the plasma membrane. A study which characterised nitrergic effects on synaptotagmins expressed synaptotagmin 1 in Hi5 insect cells and detected tyrosine nitration in 6 of 11 surface accessible tyrosine residues, three in the C2A domain (Tyr 151, Tyr 216, and Tyr 229) and three in the C2B domain (Tyr 311, Tyr 364, and Tyr 380) [67]. Although the above studies implicate that posttranslational modifications by NO have functional consequences, evidence supporting a functional role is rare. However, it is conceivable to suggest that the conformational changes induced by NO have strong impacts on protein-protein interactions of this complex release machinery. Therefore, depending on the concentration of NO and the reversibility of these posttranslational modifications, the consequences for neuronal signalling will be important and relevant in physiology and pathology.

### 2.2. Postsynapse: NO as a Modulator of Neurotransmitter Receptors

In addition to the reported presynaptic mechanisms, synaptic function also relies on the signal transmission through postsynaptic receptors. The postsynapse is a dynamic system, which can undergo potentiation and depression in response to changes in activity and demand resulting in changes in receptor expression or function [68, 69]. These synaptic plasticity phenomena can be mediated by changes in postsynaptic strength, that is, receptor densities or postsynaptic excitability in response to ion channels adaptations, and in general by changing the balance between excitation and inhibition [70]. These mechanisms are crucially involved in physiology and their disturbance contributes to pathology. Here, nitrergic posttranslational modifications can play a role in modulating these homeostatic plasticity signalling pathways by directly affecting receptor function or indirectly by modulating receptor translocation to the membrane.

#### 2.2.1. S-Nitrosylation

The most prominent postsynaptic receptor which has been shown to undergo S-nitrosylation by both exogenous and endogenous NO is the NMDAR. Specifically, the critical cysteine residue Cys 399 on the NR2A subunit is modulated leading to receptor inhibition [71] and hypoxia-induced stress conditions cause two residues on the NR1 subunit to be S-nitrosylated (Cys 744, Cys 798) further promoting channel inhibition [72]. NR1 and NR2A receptor subunits are also endogenously S-nitrosylated by prion protein signalling which requires the presence of copper. This basal and inhibitory S-nitrosylation of the NMDAR suggests a beneficial action of prion proteins, the lack of which, in prion disease, might contribute to the pathology [73]. Postsynaptic receptors, such as the NMDAR, are located within high postsynaptic density regions containing the scaffolding protein postsynaptic density-95 (PSD-95). Here, synaptic strength can be modulated, not only through direct receptor interaction but also through its localisation* via* PSDs. As such, PSD-95 has been shown to be physiologically S-nitrosylated and this modification further leads to a decreased PSD-95 clustering at synaptic sites [74].

Similarly, gephyrin is a postsynaptic scaffolding protein at the inhibitory synapse and its S-nitrosylation reduces the size of gephyrin clusters. This culminates in reduced cell surface expression of synaptic GABA_A_ receptors and thus modulates hippocampal inhibitory synaptic transmission [75]. Synaptic plasticity strongly depends on the ability of postsynaptic receptors to be incorporated in or removed from the postsynaptic membrane. Here, stargazin, which regulates AMPA receptor surface expression, is S-nitrosylated in NMDAR-dependent manner promoting binding to the AMPA receptor subunit GluR1 and thus enhances receptor surface expression [76]. Another AMPA receptor binding protein is N-ethylmaleimide sensitive factor (NSF), which, following S-nitrosylation, promotes AMPA receptor subunit GluR2 surface translocation [77]. In general, nitrergic signalling leads to a reduction in NMDAR activity, either directly by receptor or indirectly* via* PSD-95 S-nitrosylation and thus this could be interpreted as a negative feedback to reduce NO-mediated cytotoxicity. On the other hand, excitatory glutamate receptor signalling is enhanced and inhibitory GABA_A_ signalling reduced following NO activation with both modulations resulting in sufficient excitatory neurotransmission without necessarily leading to elevated NMDAR-mediated Ca^2+^ entry.

#### 2.2.2. 3-Nitrotyrosination

Although it has been shown that general nitrotyrosination of cellular proteins is increased following NO donor application or in response to certain stress conditions [78, 79], specific neuronal postsynaptic receptors as targets have not yet been identified. However, one study investigated the ultrastructural localisation of nitrotyrosine signals using immunogold labelling in untreated rat brain. The main signals were seen in the outer mitochondrial membrane, in dendritic spines, and also at synaptic vesicles in axon terminals [80], corroborating above findings related to the presynaptic release machinery. No labelling was detected specifically at postsynaptic sites; however, astrocytes showed a strong positive nitrotyrosination signal. As astrocytes are important components of the tripartite synapse and are reportedly involved in neurodegenerative conditions [81], this nitrergic modulation could be modulating the postsynaptic signalling cascade. This data suggests that physiologically, under unstimulated conditions, there is some basal nitrotyrosination present, which, due to its irreversible nature, will inevitably accumulate during aging, disease, and oxidative stress conditions. It becomes apparent that nitrergic activity can induce a variety of functional changes at the synapse and depending on the target protein and specific PTM it can modulate neuronal activities resulting in opposing effects on synaptic transmission.

## 3. Conclusions

Many neurodegenerative disorders have been associated with abnormal nitrergic signalling. In particular, enhanced levels of NO and related nitrosylation and nitrotyrosination events are evident in many cases. Several pathways in neurodegeneration by which NO has detrimental impacts on neuronal function have been reported including nitrosylation of dynamin-related protein 1 (DRP1), apolipoprotein E (ApoE), parkin, peroxiredoxin 2 (Prx2), X-linked inhibitor of apoptosis (XIAP), protein-disulphide isomerase (PDI), or glyceraldehyde-3-phosphate dehydrogenase (GAPDH) (reviewed in [10, 13]). Although nitrotyrosination seems to be much less explored there is strong evidence that this irreversible modification, which is mediated by the formation of peroxynitrite, has important contributions to pathology, particularly related to mitochondrial dysfunction [82]. Nitrotyrosination of cytochrome *c* provides an interesting example that this modification can result in a gain of protein function [83], whereas, on the other hand, nitrotyrosination can inhibit enzymes with essential tyrosine residues located in the active centre, as shown for mitochondrial MnSOD [84], a modification which is reported in several pathologies [85]. Direct evidence, for example, for the involvement of NOS-generated NO and subsequent nitrotyrosination in cell death, comes from studies using nNOS knock-out mice, in which NMDA-mediated cytotoxicity and neuronal cell death were diminished following the insult [86].

Together, there is accumulating evidence that highlights the importance of protein S-nitrosylation and nitrotyrosination in perturbing cell functions, including mitochondrial activities, protein folding, ubiquitination, synaptic transmission, and other signal transduction pathways. Alteration of one or several of these mechanisms can contribute to neuronal dysfunction and the development of neurodegenerative disorders. In this review we discussed specifically posttranslational modifications which occur directly at the synapse, including the presynaptic release machinery and postsynaptic transmitter receptor signalling. In addition to various high throughput proteomic approaches, which are essential to detect oxidative and other forms of protein modifications of general signalling molecules [87–90], it is also necessary to evaluate the physiological functional changes that occur as a result of these posttranslational modifications. The use of different model systems including mouse and* Drosophila* for degeneration as well as the knowledge of defined levels of nitric oxide as released by various donors [91] will allow further exploration of nitrergic pathways and their contributions to diseases and physiology.
